# Identification of a Susceptible and High-Risk Population for Postoperative Systemic Inflammatory Response Syndrome in Older Adults: Machine Learning–Based Predictive Model

**DOI:** 10.2196/57486

**Published:** 2024-11-22

**Authors:** Haiyan Mai, Yaxin Lu, Yu Fu, Tongsen Luo, Xiaoyue Li, Yihan Zhang, Zifeng Liu, Yuenong Zhang, Shaoli Zhou, Chaojin Chen

**Affiliations:** 1 Department of Pharmacy The Third Affiliated Hospital of Sun Yat-sen University Guangzhou China; 2 Department of Anesthesiology The Third Affiliated Hospital of Sun Yat-sen University Guangzhou China; 3 Department of Surgery and Anesthesia The Third Affiliated Hospital of Sun Yat-sen University Yuedong Hospital Meizhou China; 4 Big Data and Artificial Intelligence Center The Third Affiliated Hospital of Sun Yat-sen University Guangzhou China

**Keywords:** older adult patients, postoperative SIRS, sepsis, machine learning, prediction model

## Abstract

**Background:**

Systemic inflammatory response syndrome (SIRS) is a serious postoperative complication among older adult surgical patients that frequently develops into sepsis or even death. Notably, the incidences of SIRS and sepsis steadily increase with age. It is important to identify the risk of postoperative SIRS for older adult patients at a sufficiently early stage, which would allow preemptive individualized enhanced therapy to be conducted to improve the prognosis of older adult patients. In recent years, machine learning (ML) models have been deployed by researchers for many tasks, including disease prediction and risk stratification, exhibiting good application potential.

**Objective:**

We aimed to develop and validate an individualized predictive model to identify susceptible and high-risk populations for SIRS in older adult patients to instruct appropriate early interventions.

**Methods:**

Data for surgical patients aged ≥65 years from September 2015 to September 2020 in 3 independent medical centers were retrieved and analyzed. The eligible patient cohort in the Third Affiliated Hospital of Sun Yat-sen University was randomly separated into an 80% training set (2882 patients) and a 20% internal validation set (720 patients). We developed 4 ML models to predict postoperative SIRS. The area under the receiver operating curve (AUC), *F*_1_ score, Brier score, and calibration curve were used to evaluate the model performance. The model with the best performance was further validated in the other 2 independent data sets involving 844 and 307 cases, respectively.

**Results:**

The incidences of SIRS in the 3 medical centers were 24.3% (876/3602), 29.6% (250/844), and 6.5% (20/307), respectively. We identified 15 variables that were significantly associated with postoperative SIRS and used in 4 ML models to predict postoperative SIRS. A balanced cutoff between sensitivity and specificity was chosen to ensure as high a true positive as possible. The random forest classifier (RF) model showed the best overall performance to predict postoperative SIRS, with an AUC of 0.751 (95% CI 0.709-0.793), sensitivity of 0.682, specificity of 0.681, and *F*_1_ score of 0.508 in the internal validation set and higher AUCs in the external validation-1 set (0.759, 95% CI 0.723-0.795) and external validation-2 set (0.804, 95% CI 0.746-0.863).

**Conclusions:**

We developed and validated a generalizable RF model to predict postoperative SIRS in older adult patients, enabling clinicians to screen susceptible and high-risk patients and implement early individualized interventions. An online risk calculator to make the RF model accessible to anesthesiologists and peers around the world was developed.

## Introduction

Systemic inflammatory response syndrome (SIRS) is a nonhomeostatic, self-destructive, and uncontrollable inflammatory response of the whole body triggered by infection, trauma, or major operations [1]. Recognizing SIRS has been a prerequisite of suspecting potential sepsis and implementing decisions such as sample culturing for the source of infection, escalating antibiotic regimens, and the level of patient monitor and care [2,3]. Although there is a tendency to apply criteria including the Sequential Organ Failure Assessment (SOFA) score or quick SOFA score to identify the possibility of sepsis [4], SIRS criteria has demonstrated higher sensitivity than the quick SOFA score [3], and it has served as both useful inclusion criteria and therapeutic target of trials aiming to treat sepsis [5]. Early identification of patients who will develop postoperative SIRS may enable clinicians to provide timely interventions to prevent sepsis and improve outcomes. It has been reported that the incidence of postoperative SIRS could be as high as 89% [6,7] in patients undergoing abdominal surgery, and the condition frequently developed into sepsis and even multiple organ dysfunction syndrome [8]. A 13-fold increase in mortality was reported in patients with postoperative SIRS compared with those without SIRS [9]. Notably, the incidences of SIRS and sepsis steadily increase with age, and octogenarians are almost twice as likely to develop sepsis than those aged less than 50 years [10,11]. Although standardized preoperative antibiotic prophylaxis has been recommended and clinically applied for older adult populations with a high risk of postoperative infection and SIRS, nearly one-quarter of older adult patients still develop SIRS within 3 days after surgery [12]. Thus, it is important to identify the risk of postoperative SIRS for older adult patients at a sufficiently early stage, which would allow preemptive individualized enhanced therapy to be conducted to improve the prognosis of older adult patients.

Compared with traditional biostatistical methods, machine learning (ML) methods hold the advantages of flexibility, scalability, and the ability to analyze diverse data types, which can be deployed for many tasks, such as risk stratification, diagnosis and classification, and survival predictions [13-15]. In recent years, there have been many ML models used to predict sepsis [16-22], the majority of which were developed in similar populations such as patients in the intensive care unit [23]. However, only a few have focused on older adult surgical patients, and an ML model to predict postoperative SIRS has been rarely reported.

The goal of our study was to use ML methods to develop an individualized predictive model for older adult surgical patients to screen for a susceptible population at a high risk for SIRS in order to instruct appropriate early interventions. Meanwhile, the generalizability of the model was validated with the data sets from the other 2 medical centers.

## Methods

### Setting, Dates, and Population

The study was performed based on the electronic health record (EHR) systems of 3 independent medical centers including the Third Affiliated Hospital of Sun Yat-sen University (Guangzhou, China), Lingnan Hospital of Sun Yat-sen University, and Yuedong Hospital of Sun Yat-sen University using data from surgical patients aged ≥65 years from 2015 to 2020. 

During the retrospective enrollment, the inclusion criteria included (1) age ≥65 years, (2) patients who underwent general anesthetic with endotracheal intubation or laryngeal mask, and (3) patients who had preoperative antimicrobial prophylaxis. The exclusion criteria included (1) patients whose total intraoperative infusion volumes, fluid loss, or American Society of Anesthesiologists (ASA) classifications were not recorded and (2) patients diagnosed with SIRS before surgery or undergoing infectious surgeries.

### Data Sources

The data comes from the EHR system of the 3 hospitals, which were established by extracting medical records from the hospital information system, laboratory information system, picture archiving and communication system (PACS), and Docare Anesthesia System (2005-2020 Medical System Co, Ltd), which enabled access to a comprehensive data set collected during hospital admissions, inpatient stays, and post-hospital follow-up visits, including demographic characteristics, daily documentation, laboratory tests, imaging results, and anesthesia records.

### Outcome

SIRS was diagnosed according to the American College of Chest Physicians [24]. It was defined when 2 or more of the following criteria were present: (1) temperature <36 °C or ≥38 °C, (2) heart rate ≥90 bpm, (3) respiratory rate ≥20 bpm or arterial carbon dioxide tension <32 mm Hg, and (4) white blood cell count <4×10^9^/L, ≥12×10^9^/L, or >10% immature forms. The incidence of SIRS within 3 postoperative days was recorded in the study.

### Data Sets

Data sets from the 3 medical centers were created separately and included: (1) patient demographics such as age, gender, and smoking history; (2) preoperative complications, including diabetes, hypertension, and fever; (3) anesthesia records, including ASA, administration of ulinastatin, dexamethasone, methylprednisolone, and dexmedetomidine; (4) liquid management including volume of fluid loss, blood loss, and colloid input; (5) duration of surgery; (6) laboratory parameters, including alanine aminotransferase (ALT), white blood cell count, hemoglobin, creatinine, albumin, high-sensitivity C-reactive protein (hs-CRP), activated partial thromboplastin time, glucose, low-density lipoprotein, high-density lipoprotein, blood urea nitrogen (BUN), fibrinogen, thrombin time, lymphocyte, red blood cell count, indirect bilirubin. These variables were selected based on EHR availability and their relevance to SIRS risk according to the literature and clinical experience [25].

For the purpose of developing and validating the ML models for risk prediction, the cohort of eligible patients from the Third Affiliated Hospital of Sun Yat-sen University was randomly divided into an 80% training set (2882 patients) and a 20% internal validation set (720 patients). In addition, 2 validation data sets were created with the eligible patients from the Lingnan Hospital and Yuedong Hospital for external validation. Based on the events per variable (EPV)-10 principle [26], this study has a sufficient sample size.

### Statistical Analysis and ML Model Training

This study was reported as per the Guidelines for Developing and Reporting Machine Learning Predictive Models in Biomedical Research [27]. Categorical variables are expressed as number (%), and continuous variables that follow a normal distribution are expressed as mean (SD) or otherwise as median (IQR). The missing rates of the variables are shown in Table S1 in Multimedia Appendix 1. Missing values were imputed on each of the 3 cohorts (hospitals) independently: Categorical variables were filled in using mode counts, and continuous variables were filled in using means. The outliers were eliminated based on clinical experience, and 0 variance variables were removed.

We trained 4 different classical ML algorithms trained on the training set, including random forest (RF), XGBoost, logistic regression (LR), and multilayer perceptron (MLP) models. RF represents a bagging parallel integration algorithm, XGBoost represents a boosting algorithm; LR represents the most classical linear model, and MLP represents a neural network algorithm. Grid search was applied based on 5-fold cross-validation to find the optimal hyperparameters for each algorithm based on the training set. The formulas for the evaluation indicators in the models are provided in Multimedia Appendix 1.

The performance of the 4 algorithms was compared, and the optimal model was selected for this study. A comparison between the optimal model and nomogram we established before [28] and was also conducted with the internal validation set. Note that the prepared data sets incurred a data imbalance problem. To address this problem, we used the synthetic minority oversampling technique (SMOTE) provided by the performanceEstimation package [29,30].

The statistical analyses were all done using Python 3.7 [31-34] and R-3.6.2 [35]. All results were considered statistically significant at *P*<.05.

### Ethical Considerations

The study protocol followed the principles of the Declaration of Helsinki and was approved by the Institutional Ethics Committee of the Third Affiliated Hospital of Sun Yat-sen University on July 27, 2022 (number [2019]02-609-04). The requirements for informed consent and clinical trial registration were waived by the committee.

## Results

### Study Cohorts and Characteristics

**Figure 1:**
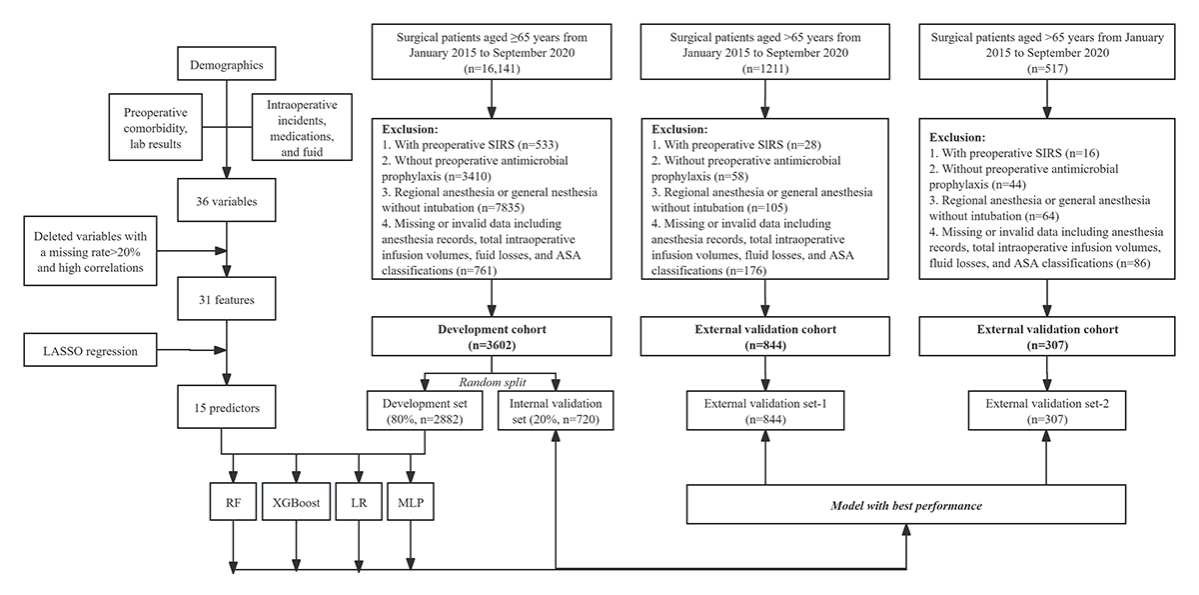
Study design and flowchart. ASA: American Society of Anesthesiologists; LASSO: least absolute shrinkage and selection operator; LR: logistic regression; MLP: multilayer perceptron; RF: random forest; SIRS: systemic inflammatory response syndrome.

Among the 16,141 patients aged ≥65 years accessed from the EHR system, only 3602 patients who met the inclusion criteria were included in the development cohort, with 876 (24.3%) postoperative SIRS events. The development cohort was then randomly separated into the training set and the internal validation set, which consisted of 2882 and 720 patients, respectively. Meanwhile, 844 and 307 patients were finally included in the external validation-1 set (Lingnan Hospital) and the external validation-2 set (Yuedong Hospital), respectively (Figure 1).

The characteristics of the 3 cohorts from different medical centers are shown in Table 1. The incidence rates of postoperative SIRS in each of the 3 medical centers were 24.3% (876/3602), 29.6% (250/844), and 6.5% (20/307), respectively. The different incidences of SIRS across the 3 medical centers may result from factors such as different regional characteristics, economic development levels, patient characteristics, surgical procedures, and the medical and nursing team.

Among the 3602 patients in the development cohort with a mean age of 70.0 years, 2144 (59.5%) were women, 2152 (59.7%) had comorbid hypertension, 1140 (31.6%) had comorbid diabetes, and 437 (12.1%) were smokers. In addition, 420 (11.7%) patients had preoperative fever, and 1330 (36.9%) were assessed as ASA III/IV/V preoperatively.

The characteristics of patients with or without postoperative SIRS in the development cohort are shown in Table 2. Patients who ended up with SIRS (SIRS group vs the non-SIRS group) were mainly women (573/876, 65.4% vs 1571/2726, 57.6%, *P*<.001) and more likely to have been diagnosed with diabetes (334/876, 38.1% vs 806/2726, 29.6%, *P*<.001) and assessed as ASA III/IV/V preoperatively (472/876, 53.9% vs 858/2726, 31.5%, *P*<.001). With evident incidence of preoperative fever (182/876, 20.8% vs 238/2726, 8.7%, *P*<.001), the total volume of fluid loss, volume of blood loss, and duration of surgery were also significantly higher and longer in the SIRS group than in the non-SIRS group (700 [330-1200] mL vs 430 [200-800] mL, 100 [50.0-200] mL vs 50 [20.0-100] mL, 207 [133-310] min vs 150 [90-232] min, respectively; all *P*<.001). Additionally, laboratory indicators including hs-CRP, BUN, direct bilirubin (DBILI), and prothrombin time (PT) were higher in SIRS patients than in non-SIRS patients, whereas albumin was lower in SIRS patients (all *P*<.001; Table 2).

**Table 1:** Baseline characteristics of patients.

Characteristics					Development cohort (n=3602)			External validation-1 (n=844)			External validation-2 (n=307)
**Postoperative SIRS** ^a^ **,** **n (%)**											
	No			2726 (75.7)			594 (70.4)			287 (93.5)	
	Yes			876 (24.3)			250 (29.6)			20 (6.5)	
**Demographics**											
	Age, (years), median (IQR)			70.0 (67.0-75.0)			70.0 (67.0-76.0)			72.0 (67.2-78.8)	
	**Gender, n (%)**										
		Female	2144 (59.5)			468 (55.5)			169 (55)		
		Male	1458 (40.5)			376 (44.5)			138 (45)		
	**Hypertension, n (%)**										
		No	1450 (40.3)			313 (37.1)			173 (56.4)		
		Yes	2152 (59.7)			531 (62.9)			134 (43.6)		
	**Diabetes, n (%)**										
		No	2462 (68.4)			526 (62.3)			51 (54.8)		
		Yes	1140 (31.6)			318 (37.7)			42 (45.2)		
	**History of smoking, n (%)**										
		No	3165 (87.9)			755 (89.5)			257 (86.2)		
		Yes	437 (12.1)			89 (10.5)			41 (13.8)		
	**Preoperative fever, n (%)**										
		No	3182 (88.3)			642 (76.1)			290 (94.5)		
		Yes	420 (11.7)			202 (23.9)			17 (5.54)		
	**ASA** ^b^ **classification, n (%)**										
		I/II	2272 (63.1)			509 (60.3)			236 (76.9)		
		III/IV/V	1330 (36.9)			335 (39.7)			71 (23.1)		
**Preoperative variables**											
	WBC^c^ (10^9^/L), median (IQR)			6.41 (5.15-8.11)			6.57 (5.38-8.66)			6.69 (5.57-8.53)	
	Lymphocytes (10^9^/L), median (IQR)			1.60 (1.20-2.05)			1.50 (1.09-1.96)			1.73 (1.32-2.19)	
	RBC^d^ (10^12^/L), median (IQR)			4.27 (3.85-4.66)			4.15 (3.74-4.62)			4.29 (3.91-4.72)	
	HGB^e^ (g/L), median (IQR)			127 (113-138)			124 (111-137)			125 (111-136)	
	RDW-CV^f^ (%), median (IQR)			0.13 (0.13-0.14)			0.13 (0.12-0.14)			13.5 (12.9-14.4)	
	hs-CRP^g^ (mg/L), median (IQR)			6.41 (5.10-8.20)			6.56 (5.30-8.66)			10.0 (4.17-34.9)	
	Albumin (g/L), median (IQR)			39.6 (36.2-42.7)			39.0 (36.0-41.7)			37.8 (34.3-40.8)	
	ALT^h^ (U/L), median (IQR)			17.0 (13.0-26.0)			18.0 (13.0-28.0)			19.0 (13.0-28.0)	
	TBILI^i^ (umol/L), median (IQR)			9.80 (7.00-13.9)			10.3 (7.30-14.7)			14.6 (11.2-19.5)	
	DBILI^j^ (umol/L), median (IQR)			3.10 (2.10-4.90)			3.51 (2.30-5.55)			3.40 (2.60-4.85)	
	IBILI^k^ (umol/L), median (IQR)			6.50 (4.50-9.10)			6.50 (4.60-9.20)			10.8 (8.15-14.4)	
	Glucose (mmol/L), median (IQR)			5.42 (4.87-6.48)			5.52 (4.83-6.69)			5.43 (4.92-6.48)	
	Creatinine (umol/L), median (IQR)			75.0 (61.0-90.0)			79.0 (64.0-96.0)			74.0 (62.4-89.4)	
	BUN^l^ (mmol/L), median (IQR)			5.67 (4.60-7.02)			5.83 (4.70-7.58)			5.66 (4.50-6.82)	
	LDL^m^ (mmol/L), median (IQR)			2.90 (2.24-3.59)			2.87 (2.24-3.53)			2.55 (2.06-2.99)	
	HDL^n^ (mmol/L), median (IQR)			1.05 (0.86-1.26)			1.11 (0.92-1.33)			1.21 (0.98-1.56)	
	PT^o^ (seconds), median (IQR)			13.2 (12.8-13.9)			13.1 (12.5-13.8)			11.1 (10.6-11.6)	
	APTT^p^ (seconds), median (IQR)			37.4 (34.9-40.0)			36.3 (33.5-39.1)			31.1 (29.2-33.7)	
	Fibrinogen (g/L), median (IQR)			3.65 (3.08-4.52)			3.67 (3.08-4.58)			3.23 (2.83-3.92)	
	TT^q^ (seconds), median (IQR)			17.5 (17.5-17.5)			17.0 (17.0-17.0)			15.7 (14.9-16.4)	
	PTINR^r^, median (IQR)			1.00 (0.96-1.06)			0.99 (0.94-1.07)			1.01 (0.96-1.05)	
**Intraoperative variables**											
	**Ulinastatin, n (%)**										
		No	2817 (78.2)			627 (74.3)			305 (99.3)		
		Yes	785 (21.8)			217 (25.7)			2 (0.65)		
	**Dexamethasone,** **n (%)**										
		No	3222 (89.5)			762 (90.3)			303 (98.7)		
		Yes	380 (10.5)			82 (9.72)			4 (1.3)		
	**Dexmedetomidine,** **n (%)**										
		No	2270 (63)			566 (67.1)			110 (35.8)		
		Yes	1332 (37)			278 (32.9)			197 (64.2)		
	Methylprednisolone (mg), median (IQR)			0.00 (0.00-0.00)			0.00 (0.00-40.0)			0.00 (0.00-40.0)	
	Total volume of fluid loss (mL), median (IQR)			500 (220-900)			505 (250-900)			420 (220-808)	
	Volume of blood loss (mL), median (IQR)			50.0 (20.0-150)			100 (30.0-200)			20.0 (10.0-80.0)	
	Intraoperative colloid (mL), median (IQR)			500 (500-500)			500 (500-500)			500 (500-500)	
	Duration of surgery (min), median (IQR)			163 (95.0-250)			170 (105-253)			140 (87.8-210)	

^a^SIRS: systemic inflammatory response syndrome.

^b^ASA: American Society of Anesthesiologists.

^c^WBC: white blood cell count.

^d^RBC: red blood cell count.

^e^HGB: hemoglobin.

^f^RDW-CV: red blood cell distribution width-coefficient of variation.

^g^hs-CRP: high sensitivity C-reactive protein.

^h^ALT: alanine aminotransferase.

^i^TBILI: total bilirubin.

^j^DBILI: direct bilirubin.

^k^IBILI: indirect bilirubin.

^l^BUN: blood urea nitrogen.

^m^LDL: low-density lipoprotein.

^n^HDL: high-density lipoprotein.

^o^PT: prothrombin time.

^p^APTT: activated partial thromboplastin time.

^q^TT: thrombin time.

^r^PTINR: international normalized ratio of prothrombin time.

**Table 2:** Characteristics of non-systemic inflammatory response syndrome (SIRS) and SIRS groups in the development cohort.

Characteristics				Development cohort							
				Total (n=3602)		Non-SIRS (n=2726)		SIRS (n=876)		*P* value	
**Demographics**											
	Age (years), median (IQR)		70.0 (67.0-75.0)		70.0 (67.0-75.0)		71.0 (67.0-76.2)		<.001		
	**Gender, n (%)**										<.001
		Female	2144 (59.5)		1571 (57.6)		573 (65.4)				
		Male	1458 (40.5)		1155 (42.4)		303 (34.6)				
	**Hypertension, n (%)**										.76
		No	1450 (40.3)		1093 (40.1)		357 (40.8)				
		Yes	2152 (59.7)		1633 (59.9)		519 (59.2)				
	**Diabetes, n (%)**										<.001
		No	2462 (68.4)		1920 (70.4)		542 (61.9)				
		Yes	1140 (31.6)		806 (29.6)		334 (38.1)				
	**History of smoking, n (%)**										.001
		No	3165 (87.9)		2423 (88.9)		742 (84.7)				
		Yes	437 (12.1)		303 (11.1)		134 (15.3)				
	**Preoperative fever, n (%)**										<.001
		No	3182 (88.3)		2488 (91.3)		694 (79.2)				
		Yes	420 (11.7)		238 (8.7)		182 (20.8)				
	**ASA** ^a^ **classification, n (%)**										<.001
		I/II	2272 (63.1)		1868 (68.5)		404 (46.1)				
		III/IV/V	1330 (36.9)		858 (31.5)		472 (53.9)				
**Preoperative variables**											
	WBC^b^ (10^9^/L), median (IQR)		6.41 (5.15-8.11)		6.32 (5.07-7.88)		6.80 (5.44-8.94)		<.001		
	Lymphocytes (10^9^/L), median (IQR)		1.60 (1.20-2.05)		1.63 (1.23-2.05)		1.54 (1.12-2.05)		.007		
	RBC^c^ (10^12^/L), median (IQR)		4.27 (3.85-4.66)		4.28 (3.88-4.66)		4.22 (3.73-4.65)		.006		
	HGB^d^ (g/L), median (IQR)		127 (113-138)		127 (115-138)		126 (110-139)		.04		
	RDW-CV^e^ (%), median (IQR)		0.13 (0.13-0.14)		0.13 (0.12-0.14)		0.13 (0.13-0.14)		<.001		
	hs-CRP^f^ (mg/L), median (IQR)		6.41 (5.10-8.20)		6.31 (5.03-7.93)		6.83 (5.46-9.20)		<.001		
	Albumin (g/L), median (IQR)		39.6 (36.2-42.7)		40.0 (36.6-42.9)		38.2 (35.0-41.7)		<.001		
	ALT^g^ (U/L), median (IQR)		17.0 (13.0-26.0)		17.0 (12.0-25.0)		19.0 (14.0-32.0)		<.001		
	TBILI^h^ (umol/L), median (IQR)		9.80 (7.00-13.9)		9.70 (6.90-13.5)		10.2 (7.30-15.2)		<.001		
	DBILI^i^ (umol/L), median (IQR)		3.10 (2.10-4.90)		3.00 (2.10-4.70)		3.50 (2.30-5.50)		<.001		
	IBILI^j^ (umol/L), median (IQR)		6.50 (4.50-9.10)		6.40 (4.60-8.90)		6.60 (4.50-9.50)		.16		
	Glucose (mmol/L), median (IQR)		5.42 (4.87-6.48)		5.41 (4.87-6.41)		5.43 (4.88-6.75)		.31		
	Creatinine (umol/L), median (IQR)		75.0 (61.0-90.0)		73.0 (60.0-88.0)		78.0 (63.0-96.0)		<.001		
	BUN^k^ (mmol/L), median (IQR)		5.67 (4.60-7.02)		5.59 (4.56-6.90)		5.96 (4.69-7.42)		<.001		
	LDL^l^ (mmol/L), median (IQR)		2.90 (2.24-3.59)		2.92 (2.26-3.59)		2.81 (2.19-3.58)		.055		
	HDL^m^ (mmol/L), median (IQR)		1.05 (0.86-1.26)		1.07 (0.88-1.28)		1.02 (0.81-1.24)		<.001		
	PT^n^ (seconds), median (IQR)		13.2 (12.8-13.9)		13.2 (12.7-13.8)		13.4 (12.9-14.2)		<.001		
	APTT^o^ (seconds), median (IQR)		37.4 (34.9-40.0)		37.2 (34.9-39.9)		38.0 (35.0-41.3)		.001		
	Fibrinogen (g/L), median (IQR)		3.65 (3.08-4.52)		3.63 (3.09-4.44)		3.77 (3.05-4.75)		.03		
	TT^p^ (seconds); median (IQR)]		17.5 (17.5-17.5)		17.5 (17.5-17.5)		17.5 (17.5-17.5)		.055		
	PTINR^q^, median (IQR)		1.00 (0.96-1.06)		1.00 (0.96-1.06)		1.02 (0.97-1.09)		<.001		
**Intraoperative variables**											
	**Ulinastatin, n (%)**										<.001
		No	2817 (78.2)		2214 (81.2)		603 (68.8)				
		Yes	785 (21.8)		512 (18.8)		273 (31.2)				
	**Dexamethasone,** **n (%)**										.81
		No	3222 (89.5)		2436 (89.4)		786 (89.7)				
		Yes	380 (10.5)		290 (10.6)		90 (10.3)				
	**Dexmedetomidine,** **n (%)**										.01
		No	2270 (63)		1749 (64.2)		521 (59.5)				
		Yes	1332 (37)		977 (35.8)		355 (40.5)				
	Methylprednisolone (mg), median (IQR)		0		0		0		<.001		
	Total volume of fluid loss (mL), median (IQR)		500 (220-900)		430 (200-800)		700 (330-1200)		<.001		
	Volume of blood loss (mL), median (IQR)		50.0 (20.0-150)		50.0 (20.0-100)		100 (50.0-200)		<.001		
	Intraoperative colloid (mL), median (IQR)		500 (500-500)		500 (500-500)		500 (500-1000)		<.001		
	Duration of surgery (min), median (IQR)		163 (95.0-250)		150 (90.0-232)		207 (133-310)		<.001		

^a^ASA: American Society of Anesthesiologists.

^b^WBC: white blood cell count.

^c^RBC: red blood cell count.

^d^HGB: hemoglobin.

^e^RDW-CV: red blood cell distribution width-coefficient of variation.

^f^hs-CRP: high sensitivity C-reactive protein.

^g^ALT: alanine aminotransferase.

^h^TBILI: total bilirubin.

^i^DBILI: direct bilirubin.

^j^IBILI: indirect bilirubin.

^k^BUN: blood urea nitrogen.

^l^LDL: low-density lipoprotein.

^m^HDL: high-density lipoprotein.

^n^PT: prothrombin time.

^o^APTT: activated partial thromboplastin time.

^p^TT: thrombin time.

^q^PTINR: international normalized ratio of prothrombin time.

The prognoses of the non-SIRS and SIRS groups in the development cohort are shown in Table 3. Compared with the non-SIRS group, patients in the SIRS group were significantly more likely to develop postoperative complications that included hemorrhage (446/876, 50.9% vs 939/2726, 34.4%, *P*<.001), acute respiratory distress syndrome (14/876, 1.6% vs 2/2726, 0.1%, *P*<.001), cardiac arrest (10/876, 1.1% vs 5/2726, 0.2%, *P*<.001), agitation and delirium (65/876, 7.4% vs 32/2726, 1.2%, *P*<.001), coma (60/876, 6.8% vs 7/2726, 0.3%, *P*<.001), and acute kidney injury (81/876, 9.2% vs 55/2726, 2%, *P*<.001). Furthermore, SIRS patients had a longer median postoperative hospitalization (12.0 [8.0-19.0] days vs 7.0 [5.0-10.0] days, *P*<.001), higher median cost (¥91,846 [¥61,765-¥135.5] vs ¥56,542 [¥34,273-¥76,956], *P*<.001; a currency exchange rate of ¥1=US $0.14 is applicable), higher risk of postoperative intensive care unit admissions (273/876, 37.1% vs 79/2726, 3.3%, *P*<.001), and higher in-hospital mortality rate (21/876, 2.4% vs 10/2726, 0.4%, *P*<.001) than non-SIRS patients.

**Table 3:** Prognoses of the non-systemic inflammatory response syndrome (SIRS) and SIRS groups in the development cohort.

Prognoses		Total cohort (n=3602)		Non-SIRS (n=2726)		SIRS (n=876)		*P* value	
**Postoperative complications, n (%)**									
	Hemorrhage	1385 (38.5)		939 (34.4)		446 (50.9)		<.001	
	ARDS^a^	16 (0.4)		2 (0.1)		14 (1.6)		<.001	
	Cardiac arrest	15 (0.4)		5 (0.2)		10 (1.1)		<.001	
	Agitation and delirium	97 (2.7)		32 (1.2)		65 (7.4)		<.001	
	Coma	67 (1.9)		7 (0.3)		60 (6.8)		<.001	
	Acute kidney injury	136 (3.8)		55 (2)		81 (9.2)		<.001	
Postoperative ICU^b^ admission, n (%)			352 (11.4)		79 (3.3)		273 (37.1)		<.001
In-hospital death, n (%)			31 (0.9)		10 (0.4)		21 (2.4)		<.001
Postoperative hospital stay (days), median (Q1-Q3)			8.0 (5.0-12.0)		7.0 (5.0-10.0)		12.0 (8.0-19.0)		<.001
Total hospital stay (days), median (Q1-Q3)			16.0 (11.0-22.0)		15.0 (10.0-20.0)		21.5 (15.0-32.0)		<.001
Total cost (¥)^c^, median (Q1-Q3)			62,434 (39,886-87,937)		56,542 (34,273-76,956)		91,846 (61,675-135,548)		<.001

^a^ARDS: acute respiratory distress syndrome.

^b^ICU: intensive care unit.

^c^A currency exchange rate of ¥1=US $0.14 is applicable.

### Variable Selection

Details of the missing rates in each center for the 36 variables selected based on the literature and our clinical experience are shown in Table S1 in Multimedia Appendix 1, and the highly correlated variables with a correlation coefficient higher than 0.7 were deleted to avoid collinearity (based on the principle of keeping the variable with the highest area under the receiver-operating curve [AUC] in the independent prediction of postoperative SIRS; Figure S1 in Multimedia Appendix 1).

**Figure 2:**
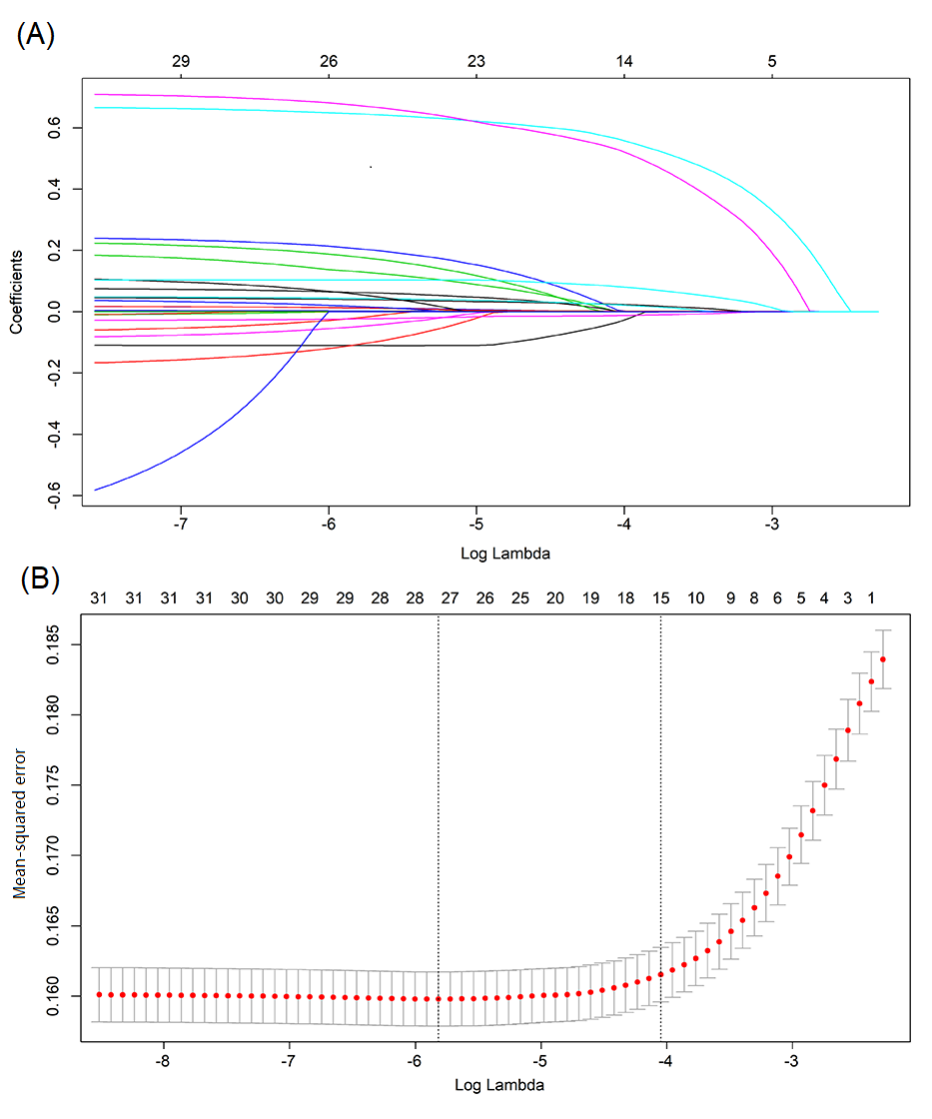
Feature selection using least absolute shrinkage and selection operator (LASSO) regression.

Since partially relevant or less important features may negatively affect the performance of ML models, feature selection was performed on the development cohort using least absolute shrinkage and selection operator (LASSO) regression methods. As shown in Figure 2, the dimensionality was reduced to 15 features after LASSO, including preoperative fever, ASA, PT, hsCRP, BUN, diabetes, duration of surgery, ulinastatin, methylprednisolone, alanine aminotransferase, total volume of fluid loss, volume of blood loss, DBILI, albumin, and gender. Except albumin and gender, which were negatively associated with postoperative SIRS, all other features were positively associated with postoperative SIRS in older adult patients (Table 4).

**Table 4:** Correlation coefficients between postoperative systemic inflammatory response syndrome (SIRS) and the 15 selected features using least absolute shrinkage and selection operator (LASSO) regression methods.

Characteristics	Correlation coefficient
Preoperative fever	0.104
ASA^a^ classification	0.101
PT^b^	0.016
hs-CRP^c^	0.005
BUN^d^	0.004
Diabetes mellitus	0.001
Duration of surgery	<0.001
Ulinastatin	<0.001
Methylprednisone	<0.001
ALT^e^	<0.001
Total volume of fluid loss	<0.001
Volume of blood loss	<0.001
DBILI^f^	<0.001
Albumin	–0.002
Gender	–0.005

^a^ASA: American Society of Anesthesiologists.

^b^PT: prothrombin time.

^c^hs-CRP: high-sensitivity C-reactive protein.

^d^BUN: blood urea nitrogen.

^e^ALT: alanine aminotransferase.

^f^DBILI: direct bilirubin.

### Model Construction, Internal Validation, and Horizontal Comparison

**Figure 3:**
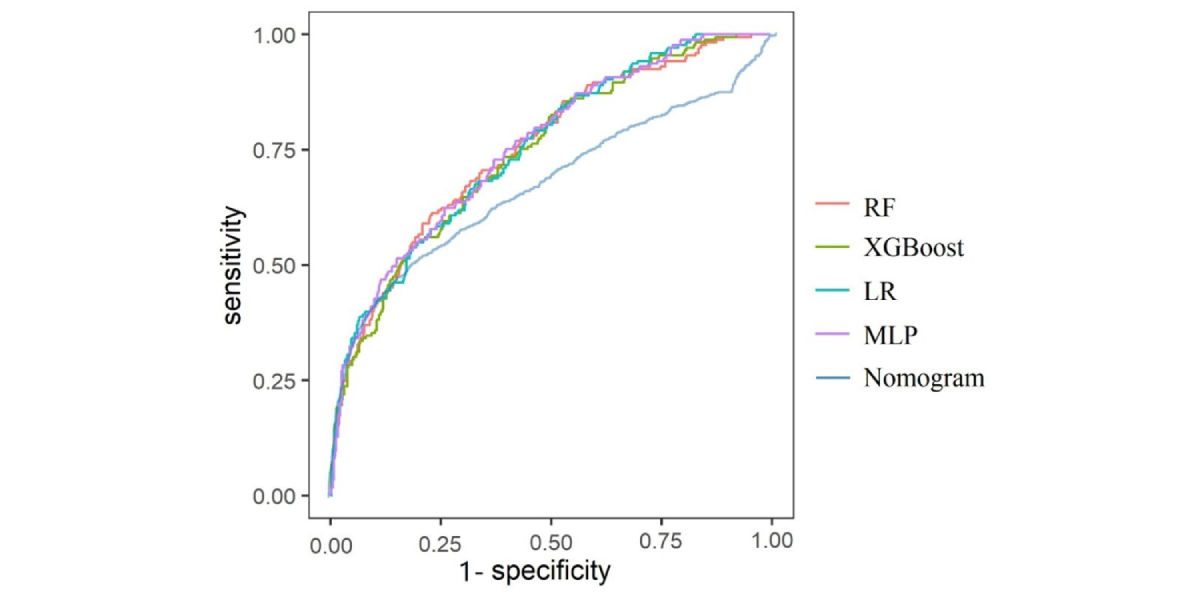
Performance of different machine learning algorithms in the internal validation set. LR: logistic regression; MLP: multilayer perceptron; RF: random forest.

**Figure 4:**
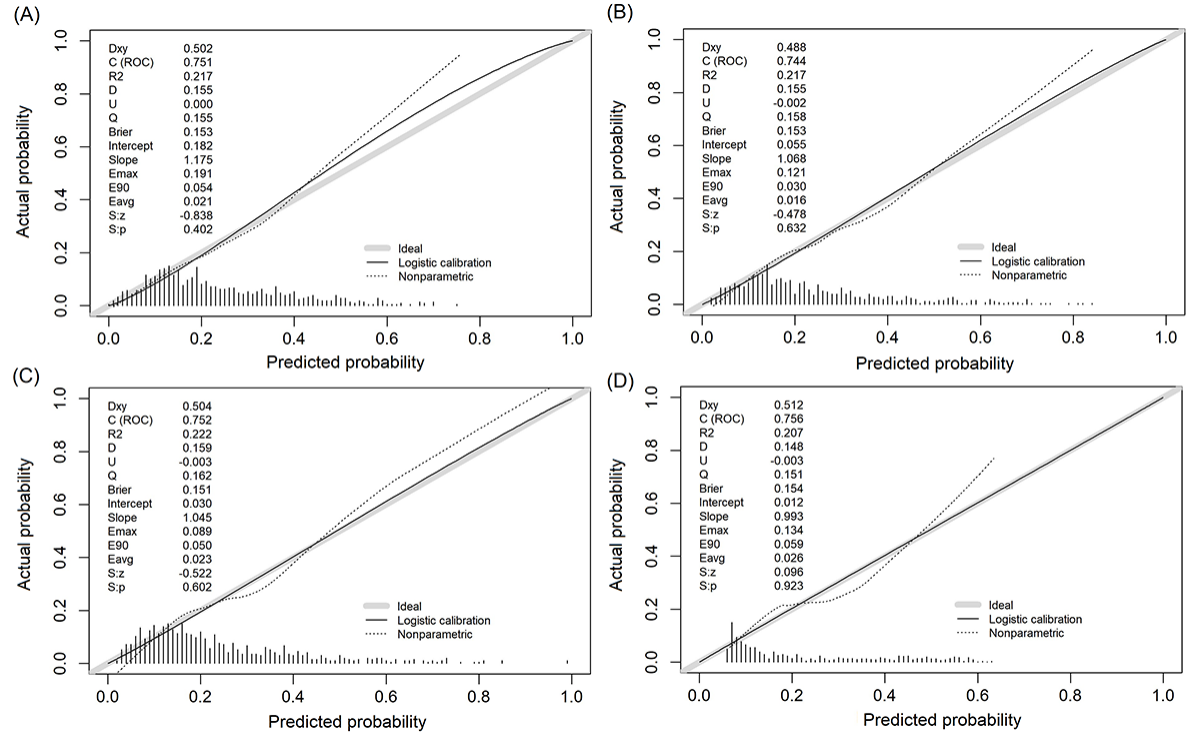
The calibration curves in the internal validation set for the (A) random forest model, (B) XGBoost model, (C) logistic regression model, and (D) multilayer perceptron model.

Finally, the 15 selected predictors were used in the 4 ML models, including RF, XGBoost, LR, and MLP, to predict postoperative SIRS. The performance of the different ML algorithms in the internal validation set are shown in Figure 3, and the calibration curves are presented in Figure 4. The AUC of the RF model was 0.751 (95% CI 0.709-0.793) with the highest sensitivity of 0.682 and specificity of 0.681. The *F*_1_ score of the RF model (0.508) was the highest among the 4 ML models, and the Brier score (0.153) was also relatively higher. As a result, upon general consideration of the AUC, *F*_1_ score, Brier score, calibration curve, and the ability of subsequent cross-center promotion and application, we thought that the RF model demonstrated the best performance. Additionally, the performance of RF model in the internal validation set was compared with the nomogram [23] we once established to predict postoperative SIRS in older patients (Figure 3). The results showed that the RF model had a significantly higher AUC (AUC=0.751) than the nomogram (AUC=0.671) in the internal validation set, which further proved the generalizability of the RF model (Table 5). The internal validation results after applying SMOTE are shown in Table S2 in Multimedia Appendix 1, where the AUC values increased for almost all ML algorithms except for LR and RF still exhibited the best performance with an AUC of 0.923.

**Table 5:** Internal validation performance of different machine learning models.

Metrics	Random forest	XGBoost	Logistic regression	Multilayer perceptron	Nomogram
AUC^a^ (95% CI)	0.751 (0.709-0.793)	0.744 (0.702-0.786)	0.752 (0.711-0.793)	0.756 (0.715-0.797)	0.671 (0.648-0.694)
Cutoff	0.237	0.226	0.230	0.216	0.276
Sensitivity	0.682	0.665	0.671	0.665	0.626
Specificity	0.681	0.667	0.672	0.665	0.625
Accuracy	0.682	0.666	0.672	0.665	0.626
*F*_1_ score	0.508	0.489	0.496	0.488	0.448
PPV^b^	0.404	0.387	0.393	0.386	0.349
NPV^c^	0.871	0.863	0.866	0.862	0.839
Brier score	0.153	0.153	0.151	0.154	0.174

^a^AUC: area under the receiver operating curve.

^b^PPV: positive predictive value.

^c^NPV: negative predictive value.

### External Validation Performance

External validation of the developed RF algorithm was conducted with the eligible data from patients from Lingnan Hospital (external validation-1) and Yuedong Hospital (external validation-2). As shown in Table 6, the RF model achieved relatively higher AUCs for the external validation-1 (0.759, 95% CI 0.723-0.795) and external validation-2 (0.804, 95% CI 0.746-0.863) sets. Moreover, the external validation-2 set demonstrated much higher sensitivity (lower false-negative rate) than the internal validation set (0.800 vs 0.682), and the specificity of the model was improved in both external validation sets. On the other hand, the negative predictive value was high, and the positive predictive value (PPV) was relatively low for all 3 validation sets since we chose a balanced cutoff between sensitivity and specificity to ensure as high a true positive (TP) as possible, which also results in a high false positive (FP) and low false negative. Notably, the PPV and *F*_1_ score of external validation-2 was very low due to the low incidence rate of postoperative SIRS (20/307, 6.5%) at Yuedong Hospital.

After applying SMOTE, the performance of the RF model in the external validation set was basically the same as the model without SMOTE, with AUCs of 0.783 vs 0.759 in external validation set 1 and 0.784 vs 0.804 in external validation set 2, which further proves that the RF model has good generalizability to different validation populations and different data processing methods (Table S3 in Multimedia Appendix 1).

**Table 6:** External validation performance of the random forest model.

Metrics	Internal validation	External validation-1	External validation-2
AUC^a^ (95% CI)	0.751 (0.709-0.793)	0.759 (0.723-0.795)	0.804 (0.746-0.863)
Cutoff^b^	0.237	0.237	0.237
Sensitivity	0.682	0.680	0.800
Specificity	0.681	0.689	0.690
Accuracy	0.682	0.686	0.697
*F*_1_ score	0.508	0.562	0.256
PPV^c^	0.404	0.479	0.152
NPV^d^	0.871	0.836	0.980

^a^AUC: area under the receiver operating curve.

^b^We chose a cutoff balanced between sensitivity and specificity.

^c^PPV: positive predictive value.

^d^NPV: negative predictive value.

### Feature Importance Weight

**Figure 5:**
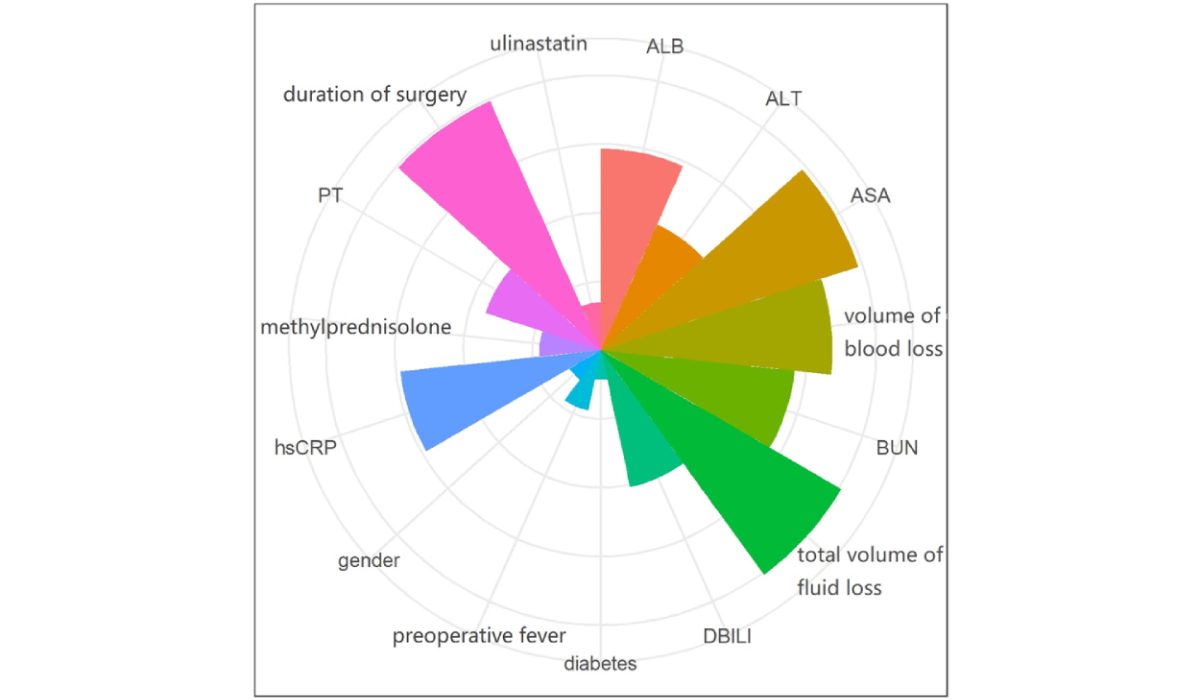
Feature importance weight of the random forest algorithm. ALB: albumin; ALT: alanine aminotransferase; ASA: American Society of Anesthesiologists; BUN: blood urea nitrogen; DBILI: direct bilirubin; hsCRP: high sensitivity C-reactive protein; PT: prothrombin time.

The feature importance permutation was used to rank the levels of feature importance, which is defined as the decrease in a model score when a single feature value is randomly shuffled (Figure 5). The results showed that the total volume of fluid loss, duration of surgery, ASA, and volume of blood loss had a significant impact on the outcome, with higher importance weight values of 0.121, 0.119, 0.118, and 0.101, respectively (Table 7).

**Table 7:** Feature importance weight of the random forest algorithm.

Characteristics	Permutation importance scores, mean (SD)	Feature importance weight
Total volume of fluid loss	0.047 (0.002)	0.121
Duration of surgery	0.047 (0.001)	0.119
ASA^a^	0.046 (0.003)	0.118
Volume of blood loss	0.039 (0.001)	0.101
hs-CRP^b^	0.035 (0.002)	0.088
Albumin	0.034 (0.002)	0.088
BUN^c^	0.033 (0.000)	0.085
DBILI^d^	0.024 (0.001)	0.061
ALT^e^	0.023 (0.001)	0.060
PT^f^	0.021 (0.001)	0.053
Methylprednisolone	0.011 (0.001)	0.027
Preoperative fever	0.011 (0.001)	0.027
Ulinastatin	0.008 (0.001)	0.021
Gender	0.006 (0.000)	0.016
Diabetes mellitus	0.005 (0.000)	0.013

^a^ASA: American Society of Anesthesiologists.

^b^hs-CRP: high sensitivity C-reactive protein.

^c^BUN: blood urea nitrogen.

^d^DBILI: direct bilirubin.

^e^ALT: alanine aminotransferase.

^f^PT: prothrombin time.

### Online Application

Figure S2 in Multimedia Appendix 1 shows the online calculator for our RF model to predict the risk of postoperative SIRS in older adult patients, which can be found at [36]. Users can quickly obtain the predicted probability of the patient’s postoperative SIRS risk by entering the values of the 15 predictors.

## Discussion

### Principal Findings

In this study, we evaluated the ability of 4 ML algorithms including RF, XGBoost, LR, and MLP to predict postoperative SIRS in older adult patients based on eligible data from EHR systems and concluded that the RF model has moderately better performance than the other ML algorithms, with an AUC value of 0.751, highest sensitivity of 0.682, and specificity of 0.681. The RF model also exhibited better performance in the internal validation set than the nomogram we once established to predict postoperative SIRS in older patients. Furthermore, the applicability of the RF model was proved by external validation in the other 2 independent medical centers, with relatively higher AUC values of 0.759 and 0.804, indicating good reproducibility and generalizability of the model in older adult patients. Notably, the model exhibited a high negative predictive value and relatively low PPV because we chose a balanced cutoff between sensitivity and specificity to ensure as high a TP as possible, which inevitably leads to a high FP and low false negative. Especially for external validation-2 (Yuedong Hospital), which had a very low incidence rate of postoperative SIRS (20/307, 6.5%), 16 TP were successfully predicted with a high sensitivity of 0.800, which comes with 89 FP and results in a low PPV (0.152) and *F*_1_ score (0.256).

The model indicators used in this study include preoperative fever, ASA, PT, hsCRP, BUN, diabetes, duration of surgery, ulinastatin, methylprednisolone, ALT, total volume of fluid loss, volume of blood loss, DBILI, albumin, and gender. These indicators are mainly related to the individual disease characteristics and are not affected by the level of different medical institutions or regional characteristics, which makes the model more generalizable. The results of our study further prove that the model has a good generalization ability for populations with different SIRS incidences. The high recall of our model is important for reminders to check patients early and prevent SIRS in advance; this demonstrates a more responsible attitude toward patients.

Currently, given the more severe mortality and adverse prognosis, most studies have adopted sepsis as a clinical endpoint [2]. In our study, we used SIRS as the primary outcome because it has been an acknowledged criterion that is easier to identify and can help physicians notice the possibility of sepsis and prescribe tests to examine whether infection truly exists.

As the population ages, the surgical population is also aging faster than the general population, with higher morbidity and mortality rates [37]. Notably, older adults are predisposed to postoperative infections, SIRS, and even sepsis due to preexisting comorbidities, repeated and prolonged hospitalizations, immune dysregulation, and functional limitations [38]. Although preoperative antimicrobial prophylaxis has been routinely administered for older adult patients in various specialized operations, the persistent high incidence of postoperative SIRS suggests that it cannot be effectively prevented in this way [12]. In fact, for older patients undergoing different types of surgery, there is still a lack of effective tools to identify high-risk patients with postoperative SIRS and assist with decision-making on the need for individualized interventions.

In this study, we used ML methods to analyze diverse data types [13] due to the advantages of flexibility and scalability. A predictive model based on an RF algorithm was developed to identify older adult patients with a high risk of postoperative SIRS. Internal validation and two external validations confirmed that the established model could predict postoperative SIRS with high accuracy and specificity. The results might be important derivatives and supplements to the current perioperative prevention and management programs, which enable surgeons and anesthesiologists to identify older adult patients who are suspected of having postoperative SIRS. In addition to screening high-risk groups, this model can also help prevent and treat postoperative SIRS in older adult patients more accurately and in a timelier manner by using various drug or nondrug means under the guideline of enhanced recovery after surgery, so as to promote the short-term and long-term prognoses of patients [39]. Finally, an online risk calculator was developed to improve clinical usability and make our model accessible to anesthesiologists and peers around the world.

### Comparison With Prior Work

Distinguished from previous investigations that mainly focused on a single indicator or a single surgery, we fully used the perioperative data of patients and constructed an optimal combination of risk factors to predict postoperative SIRS in older adult patients. In this study, 15 variables were identified to be significantly associated with postoperative SIRS, including preoperative fever, ASA [40], PT, hsCRP, BUN, diabetes [41], duration of surgery [42], ulinastatin, methylprednisolone, ALT, total volume of fluid loss, volume of blood loss, DBILI [43], albumin, and gender [44]. These variables have also been reported to be associated with postoperative SIRS and sepsis in earlier studies, adding clinical credibility to our model. Meanwhile, all the variables were routinely recorded and widely used in clinical practice, which makes the model more feasible and can be widely used in different hospitals.

Methylprednisolone and ulinastatin have also been associated with postoperative SIRS, which may be attributed to the fact that both medicines are widely used among high-risk surgical patients with postoperative infection according to anesthesiologists’ clinical experience [45-47]. Additionally, intraoperative fluid loss ranks first in the feature importance weight of the RF model, which indicates that fluid management such as intraoperative intravenous infusion should be given higher priority during the perioperative period to prevent postoperative SIRS, and this was consistent with the consensus on early management of sepsis [48].

### Limitations

Notably, several limitations must be listed here, including that (1) this is a retrospective cohort study with collection and entry bias, as well as possible residual confounding, which requires future prospective studies to validate the model. In addition, some indicators for SOFA scoring were missing, so we could not perform a comparative study between our model and SOFA scoring. (2) We selected 36 variables based on the availability in the EHR, correlation with SIRS risk, and predictive potential, but it should be noted that we may have missed other indicators with more predictive performance in postoperative SIRS due to the limitation of our data. (3) The model demonstrated a low PPV because we wanted the model to have a relatively high recall rate to predict more patients who were truly at a high risk of postoperative SIRS, which would inevitably lead to more FPs, but from a health economics perspective, the economic burden associated with enhanced perioperative management of high-risk patients is much less than that resulting from the progression of the patient to SIRS or even after sepsis, resulting in medical-economic stress. (4) The generalizability of this model in different ethnic groups and different regions also needs further validation. (5) It should be emphasized that estimates in our model are predictive and should not be interpreted as causal [49], such as the association between intraoperative use of methylprednisolone and ulinastatin and the higher SIRS incidence rate. Intraoperative use of such drugs is likely a marker of risk stratification with a lower risk of infection patients using these drugs than those at high risk.

### Conclusions

We enrolled 3 independent cohorts to develop and validate a generalizable RF model for the prediction of postoperative SIRS in older adult patients that enables surgeons and anesthesiologists to screen susceptible and high-risk populations for SIRS in older adult surgical patients and to implement early individualized interventions based on existing prevention and management programs.

## Appendix

Multimedia Appendix 1 Missing rates, formulas for evaluation indicators, deleted variables, internal validation results after applying SMOTE, external validation, and the online calculator.

## Abbreviations

ALT: alanine aminotransferase
ASA: American Society of Anesthesiologists
AUC: area under the receiver operating curve
BUN: blood urea nitrogen
DBILI: direct bilirubin
EHR: electronic health record
EPV: events per variable
FP: false positive
hs-CRP: high-sensitivity C-reactive protein
LASSO: least absolute shrinkage and selection operator
LR: logistic regression
ML: machine learning
MLP: multilayer perceptron
PACS: picture archiving and communication system
PPV: positive predictive value
PT: prothrombin time
RF: random forest
SIRS: systemic inflammatory response syndrome
SMOTE: synthetic minority oversampling technique
SOFA: Sequential Organ Failure Assessment
TP: true positive

## Data Availability

The raw data supporting the conclusions of this article will be made available by the corresponding authors via email.
