# Supplementary material for: Identification of a Susceptible and High-Risk Population for Postoperative Systemic Inflammatory Response Syndrome in Older Adults: Machine Learning–Based Predictive Model
Source: J Med Internet Res. 2024 Nov 22;26:e57486. doi: 10.2196/57486 (PMC11624453; doi:10.2196/57486)
Supplement: Multimedia Appendix 1 [file jmir_v26i1e57486_app1.docx]

## Supplementary materials

**Identification of a Susceptible and High-Risk Population for Postoperative Systemic Inflammatory Response Syndrome in Older Adults: Machine Learning–Based Predictive Model**

Haiyan Mai^1,*^; Yaxin Lu^2,*^; Yu Fu^1,*^; Tongsen Luo^3^; Xiaoyue Li^3^; Yihan Zhang^3^; Zifeng Liu^2^; Yuenong Zhang^4^; Shaoli Zhou^3^; Chaojin Chen^2,3^

**Supplementary methods**

We evaluated each model in terms of accuracy, precision, recall, and F1-score. These parameters are defined as follows:

1. Sensitivity = True positives / ( True positives + False negatives)
2. Specificity = True negatives / ( True negatives + False positives)
3. Accuracy = (True positive + True negatives) / (True positive + True negatives + False positives + False negatives)
4. Precision = True positives / ( True positives + False positives)
5. Recall = True positives / ( True positives + False negatives)
6. F1 score = 2 * P * R / (P + R)
7. PPV = True positives / ( True positives + False positives)
8. NPV = True negatives / ( True negatives + False negatives)

In addition, AUC curve was drawn to compare the performance of each model.

**Supplementary results**

**Supplementary tables**

Table S1. Missing rates of variables.

| **Characteristics** | **Development cohort**  **n=3602** | **External validation-1 n=844** | **External validation-2 n=307** |
| --- | --- | --- | --- |
| **Demographics** |  |  |  |
| Age | 0 (0%) | 0 (0%) | 1 (0.3%) |
| Gender | 0 (0%) | 0 (0%) | 0 (0%) |
| Hypertension | 0 (0%) | 0 (0%) | 0 (0%) |
| Diabetes | 0 (0%) | 0 (0%) | 0 (0%) |
| History of smoking | 0 (0%) | 0 (0%) | 9 (2.9%) |
| Preoperative fever | 0 (0%) | 0 (0%) | 0 (0%) |
| ASA classification | 0 (0%) | 0 (0%) | 0 (0%) |
| **Preoperative variables**^a^ |  |  |  |
| WBC | 135 (3.7%) | 25 (3.0%) | 6 (2.0%) |
| LYM | 251 (7.0%) | 57 (6.8%) | 6 (2.0) |
| RBC | 135 (3.7%) | 25 (3.0%) | 6 (2.0%) |
| HGB | 152 (4.2%) | 30 (3.6%) | 6 (2.0%) |
| RDW-CV | 191 (5.3%) | 51 (6.0%) | 6 (2.0%) |
| hs-CRP | 181 (5.0%) | 34 (4.0%) | 138 (45.0%) |
| Albumin | 76 (2.1%) | 8 (0.9%) | 12 (3.9%) |
| ALT | 172 (4.8%) | 32 (4.0%) | 12 (3.9%) |
| TBILI | 339 (9.4%) | 61 (7.2%) | 12 (3.9%) |
| DBILI | 340 (9.4%) | 61 (7.2%) | 12 (3.9%) |
| IBILI | 335 (9.3%) | 61 (7.2%) | 12 (3.9%) |
| GLU | 152 (4.2%) | 22 (2.6%) | 10 (3.3%) |
| Creatinine | 162 (4.5%) | 30 (3.6%) | 6 (2.0%) |
| BUN | 162 (4.5%) | 30 (3.6%) | 6 (2.0%) |
| LDL | 526 (14.6%) | 172 (20.4%) | 53 (17.3%) |
| HDL | 527 (14.6%) | 172 (20.4%) | 53 (17.3%) |
| PT | 163 (4.5%) | 67 (7.9%) | 23 (7.5%) |
| APTT | 183 (5.1%) | 42 (5.0%) | 23 (7.5%) |
| FIB | 183 (5.1%) | 42 (5.0%) | 23 (7.5%) |
| TT | 192 (5.3%) | 402 (47.6%) | 23 (7.5%) |
| PTINR | 191 (5.3%) | 41 (4.9%) | 23 (7.5%) |
| **Intraoperative variables** |  |  |  |
| Ulinastatin | 0 (0%) | 0 (0%) | 0 (0%) |
| Dexamethasone | 0 (0%) | 0 (0%) | 0 (0%) |
| Dexmedetomidine | 0 (0%) | 0 (0%) | 0 (0%) |
| Methylprednisolone | 0 (0%) | 0 (0%) | 1 (0.3%) |
| Total volume of fluid loss | 37 (1.0%) | 11 (1.3%) | 0 (0%) |
| Volume of blood loss | 34 (0.9%) | 11 (1.3%) | 0 (0%) |
| Intraoperative colloid | 34 (0.9%) | 11 (1.3%) | 0 (0%) |
| Duration of surgery | 220 (6.1%) | 69 (8.2%) | 5 (1.6%) |

^a^WBC, White blood cell count; LYM, Lymphocyte; RBC, Red blood cell count; HGB, Hemoglobin; RDW-CV, Red blood cell distribution width- coefficient of variation; hs-CRP, high sensitivity C-reactive protein; ALT, Alanine aminotransferase; TBILI, Total bilirubin; DBILI, Direct bilirubin; IBILI, Indirect bilirubin; GLU, Glucose; BUN, Blood urea nitrogen; LDL, Low density lipoprotein; HDL, High density lipoprotein; PT, prothrombin time; APTT, activated partial thromboplastin time; FIB, Fibrinogen; TT, Thrombin time; PTINR, international normalized ratio of prothrombin time

Table S2. Performance of different ML algorithms developed by SMOTE.

| **Metrics**^a^ | **SMOTE-RF** | **SMOTE-XGBoost** | **SMOTE-LR** | **SMOTE-MLP** |
| --- | --- | --- | --- | --- |
| AUC | 0.923 (0.904,0.942) | 0.895 (0.872,0.918) | 0.736 (0.699,0.773) | 0.822 (0.791,0.853) |
| Cutoff^a^ | 0.465 | 0.505 | 0.454 | 0.434 |
| Sensitivity | 0.844 | 0.816 | 0.666 | 0.762 |
| Specificity | 0.844 | 0.816 | 0.666 | 0.763 |
| Accuracy | 0.844 | 0.816 | 0.666 | 0.763 |
| F1 score | 0.843 | 0.814 | 0.664 | 0.748 |
| PPV | 0.842 | 0.813 | 0.662 | 0.735 |
| NPV | 0.847 | 0.818 | 0.67 | 0.788 |

^a^AUC, Area under curve; PPV, Positive predictive value; NPV, Negative predictive value

Table S3. External validation performance of RF model developed by SMOTE.

| **Metrics**^a^ | **Internal validation** | **External validation-1** | **External validation-2** |
| --- | --- | --- | --- |
| AUC | 0.923 (0.904,0.942) | 0.783 (0.756,0.811) | 0.784 (0.756,0.812) |
| Cutoff^a^ | 0.465 | 0.465 | 0.465 |
| Sensitivity | 0.844 | 0.776 | 0.784 |
| Specificity | 0.844 | 0.594 | 0.59 |
| Accuracy | 0.844 | 0.685 | 0.687 |
| F1 score | 0.843 | 0.711 | 0.715 |
| PPV | 0.842 | 0.657 | 0.657 |
| NPV | 0.847 | 0.726 | 0.732 |

^a^AUC, Area under curve; PPV, Positive predictive value; NPV, Negative predictive value

**Supplementary figure**


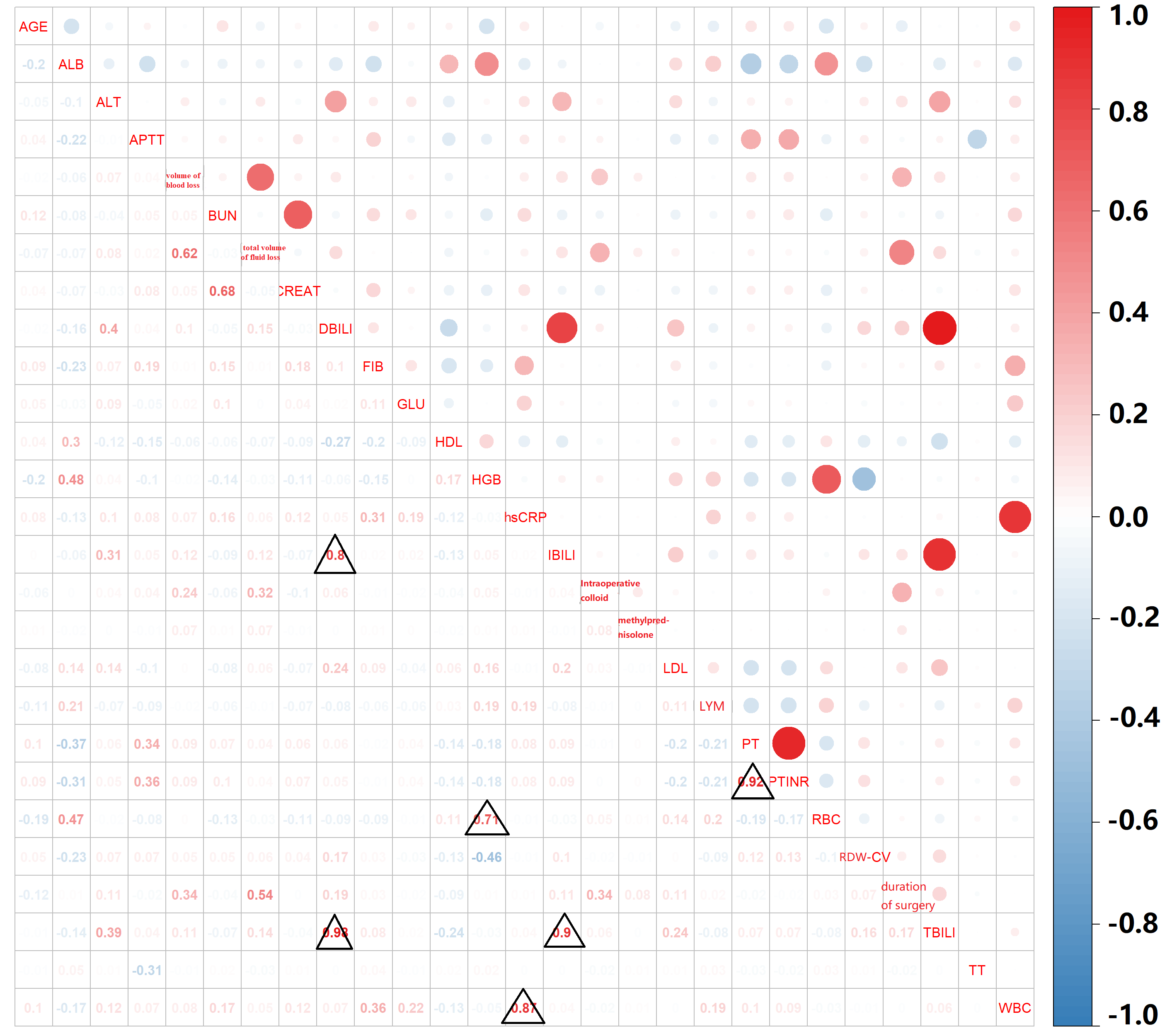


Figure S1. Colinearity analysis among the variables in the study.


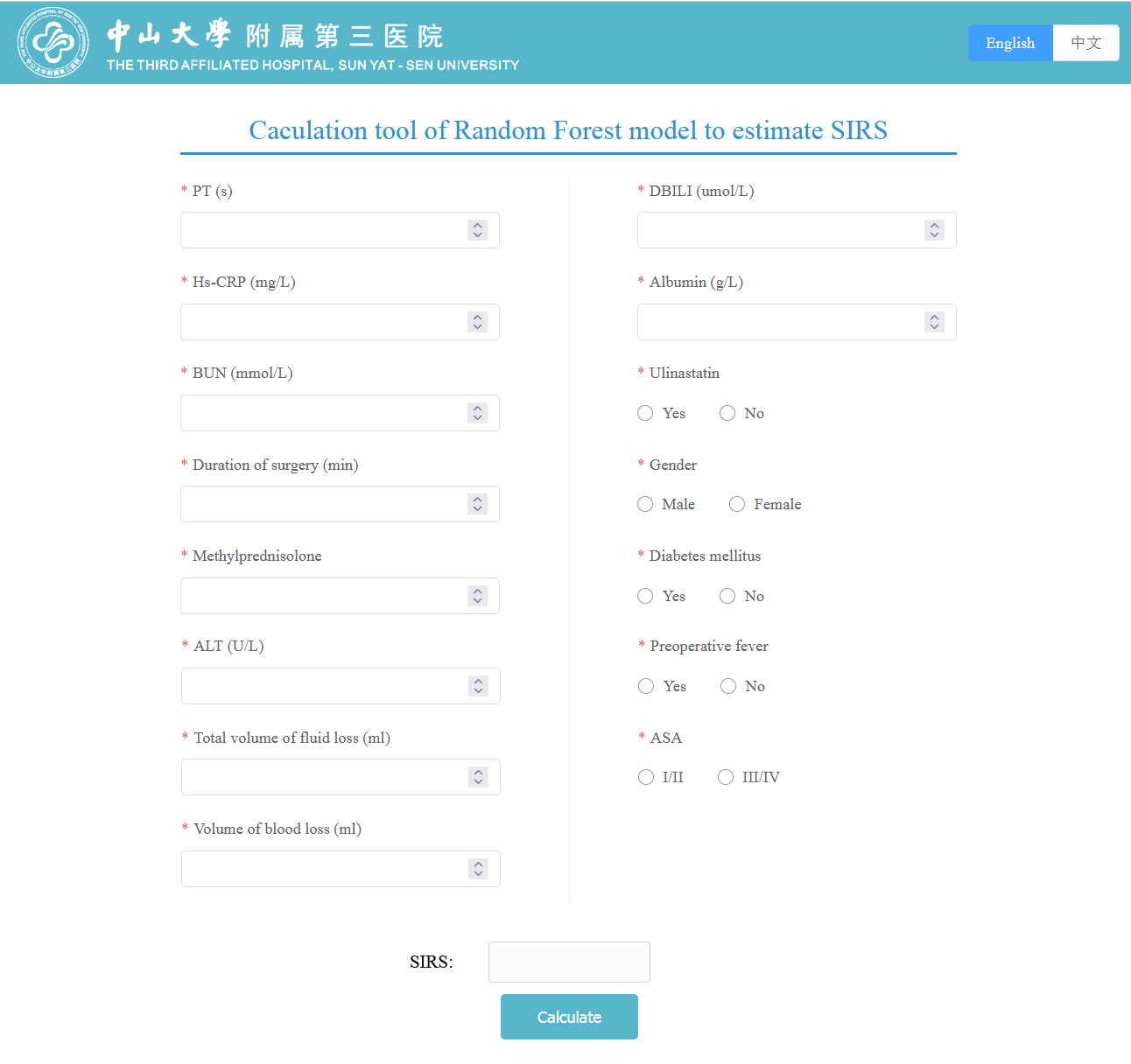


Figure S2. Online calculator of our RF model for predicting the risk of postoperative SIRS in the elderly patients.
